# TGF-β mediates aortic smooth muscle cell senescence in Marfan syndrome

**DOI:** 10.18632/aging.101998

**Published:** 2019-05-30

**Authors:** Wei You, Yimei Hong, Haiwei He, Xiaoran Huang, Wuyuan Tao, Xiaoting Liang, Yuelin Zhang, Xin Li

**Affiliations:** 1 The Second School of Clinical Medicine, Southern Medical University, Guangzhou, Guangdong 510515, China; 2 Department of Emergency Medicine, Department of Emergency and Critical Care Medicine, Guangdong Provincial People's Hospital, Guangdong Academy of Medical Sciences, Guangzhou, Guangdong 510080, China; 3 Clinical Translational Medical Research Center, Shanghai East Hospital, Tongji University School of Medicine, Shanghai, 200120, China

**Keywords:** transforming growth factor β, Marfan syndrome, vascular smooth muscle cells, senescence, reactive oxygen species

## Abstract

Formation of aortic aneurysms as a consequence of augmented transforming growth factor β (TGF-β) signaling and vascular smooth muscle cell (VSMC) dysfunction is a potentially lethal complication of Marfan syndrome (MFS). Here, we examined VSMC senescence in patients with MFS and explored the potential mechanisms that link VSMC senescence and TGF-β. Tissue was harvested from the ascending aorta of control donors and MFS patients, and VSMCs were isolated. Senescence-associated β-galactosidase (SA-β-gal) activity and expression of senescence-related proteins (p53, p21) were significantly higher in aneurysmal tissue from MFS patients than in healthy aortic tissue from control donors. Compared to control-VSMCs, MFS-VSMCs were larger with higher levels of both SA-β-gal activity and mitochondrial reactive oxygen species (ROS). In addition, TGF-β1 levels were much higher in MFS- than control-VSMCs. TGF-β1 induced VSMC senescence through excessive ROS generation. This effect was suppressed by Mito-tempo, a mitochondria-targeted antioxidant, or SC-514, a NF-κB inhibitor. This suggests TGF-β1 induces VSMC senescence through ROS-mediated activation of NF-κB signaling. It thus appears that a TGF-β1/ROS/NF-κB axis may mediate VSMC senescence and aneurysm formation in MFS patients. This finding could serve as the basis for a novel strategy for treating aortic aneurysm in MFS.

## INTRODUCTION

Marfan syndrome (MFS) is a connective tissue disorder that affects multiple organ systems, including the cardiovascular, skeletal and ocular systems. It is mainly caused by mutations in the gene that encodes fibrillin-1 (FBN1), the major component of extracellular microfibrils [1, 2]. Aortic root dilatation and aortic aneurysms or dissection are the major causes of death in MFS patients. It has been well documented that dysfunctional FBN1-induced fragmentation of microfibrils leads to activation of transforming growth factor-beta (TGF-β) signaling, and that TGF-β is closely linked to the development of aortic aneurysms in patients with MFS [3, 4]. The underlying mechanism by which TGF-β mediates aneurysm formation is not fully understood, however.

Vascular smooth muscle cells (VSMCs) are the major cell type in the tunica media of blood vessel walls and are key players in the regulation blood pressure and flow. VSMCs also maintain the matrix components of the media, and their dysfunction results in remodeling of the aortic wall [5]. For example, VSMC dysfunction reportedly plays an important role in development and progression of aortic aneurysms [6, 7]. Inducible by various stressors, cellular senescence is irreversible proliferative arrest that is closely associated with diverse age-related diseases, including those of the cardiovascular system [8, 9]. VSMC senescence has been shown to contribute to aortic aneurysm formation through release of proinflammatory cytokines and other matrix-degrading molecules [10]. Ablation of the antisenescence gene SIRT1 in VSMCs promotes aortic aneurysm formation induced by Ang II in Apoe^−/−^ mice, while overexpression of SIRT1 in VSMCs has the opposite effect. This suggests that VSMC senescence contributes to the pathogenesis in aortic aneurysm [11], though its involvement in aneurysm formation in MFS patients remains largely unknown.

Although the potential mechanisms underlying cellular senescence are not yet fully understood, it is known that elevated levels of reactive oxygen species (ROS) are associated with the induction of cellular senescence [10, 12]. NF-κB activation also appears to signal induction of cellular senescence in various cell types, including VSMCs [13, 14]. Moreover, increasing ROS levels can activate NF-κB signaling to release senescence-associated secretory phenotype (SASP) factors, which, in turn, further stimulate cellular senescence [15]. However, it remains to be determined whether TGF-β induces VSMC senescence via ROS/NF-κB signaling in MFS patients. In the present study, therefore, we examined the involvement of TGF-β1 and ROS/NF-κB signaling in VSMC senescence in MFS patients.

## RESULTS

### Increased VSMC senescence in aortic aneurysmal tissue from MFS patients

**Figure 1:**
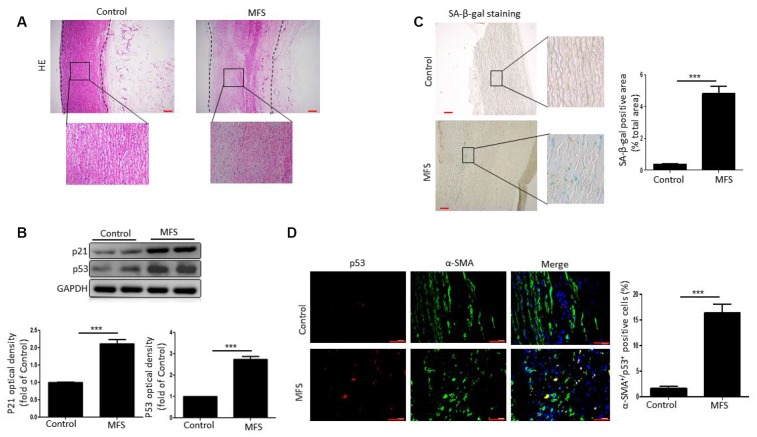
**VSMCs exhibit senescence in aortic aneurysm tissue from MFS patients.** (**A**) Representative images of HE stained sections of ascending aorta from control donors and aortic aneurysm from MFS patients. Note the degeneration of the medial layer of the aortic wall in MFS patients. The box shows the location of the magnified region. Scale bar=200 μm. (**B**) Western blot and quantitative analysis of p53 and p21 levels in the ascending aorta of control donors and MFS patients. (**C**) Representative images and quantitative analysis of SA-β-gal staining in the ascending aorta from control donors and MFS patients. The box shows the location of the magnified region. Scale bar=200 μm. (**D**) Representative images and quantitative analysis of p53 (red) and α-SMA (green) staining in the ascending aorta from control donors and MFS patients. Scale bar=50 μm. Data are expressed as the mean±SEM; n=6. ***p<0.001.

We aimed to determine whether there is a link between VSMC senescence and formation of aortic aneurysms in patients with MFS. Hematoxylin/eosin (HE)-stained aortic tissue sections from patients with MFS displayed the typical characteristics of aneurysm, including increased dilation and degeneration of the medial layer of the aorta (Figure 1A).

We next examined cellular senescence within the aortic aneurysm of MFS patients. Western blotting revealed that levels of the cellular senescence markers p53 and p21 were significantly higher in aortic tissue from MFS patients than control donors (Figure 1B). Likewise, staining revealed that senescence-associated β-galactosidase (SA-β-gal) activity was also significantly elevated in MFS patients, and that the increased SA-β-gal positivity was mainly localized to medial VSMCs (Figure 1C). The further assess the involvement of VSMC senescence in aortic aneurysms in MFS patients, we double stained aortic tissue samples for the VSMC marker α-SMA and the cellular senescence marker p53. We found that the numbers of α-SMA^+^p53^+^ double-positive cells were markedly higher in tissues from MFS patients than control donors (Figure 1D). Collectively, these findings suggest that VSMCs are senescent in aortic aneurysmal tissue from MFS patients.

### VSMCs isolated from MFS patients exhibit cellular senescence

**Figure 2:**
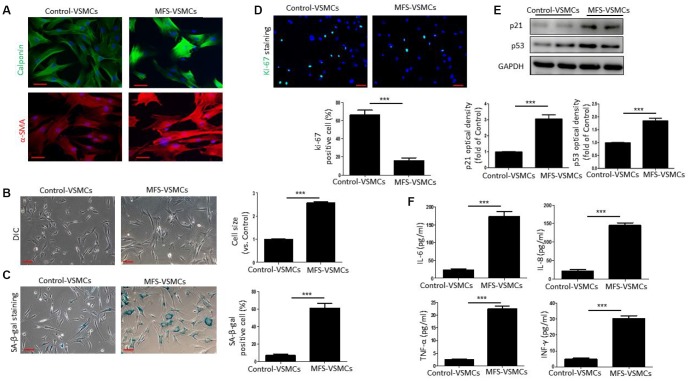
**VSMCs isolated from the ascending aorta of MFS patients exhibit cellular senescence.** (**A**) Representative images of immunofluorescent staining for α-SMA and calponin in control- and MFS-VSMCs. Scale bar=50 μm. (**B**) Representative light micrographs of control- and MFS-VSMCs. Cell size is expressed relative to control. Scale bar=100 μm. (**C**) Representative images and quantitative analysis of SA-β-gal staining in control- and MFS-VSMCs. Numbers of SA-β-gal-positive cells are expressed as percentages of the total numbers of control- or MFS-VSMCs. Scale bar=100 μm. (**D**) Representative images and quantitative analysis of immunofluorescent ki-67 staining in control- and MFS-VSMCs. Numbers of ki-67-positive cells are expressed as percentages of the total numbers of control- or MFS-VSMCs. Scale bar=100 μm. (**E**) Western blotting and quantitative analysis of p53 and p21 levels in control- and MFS-VSMCs. (**F**) Concentrations of IL-6, IL-8, TNF-α and INF-γ in medium conditioned by control- or MFS-VSMCs. Data are expressed as the mean±SEM. n=3. ***p<0.001.

To further examine VSMC senescence in MFS patients, we first isolated VSMCs from aortic tissue from MFS patients and control donors. Both control-VSMCs and MFS-VSMCs expressed α-SMA and calponin (Figure 2A), indicating that VSMCs had been successfully isolated. As shown in Figure 2B, control-VSMCs had a healthy spindle shape, whereas MFS-VSMCs were greatly enlarged and flattened (Figure 2B). In addition, the frequency of SA-β-gal positivity was significantly higher among MFS-VSMCs than control-VSMCs (Figure 2C). By contrast, numbers of ki-67-positive cells was dramatically lower in MFS-VSMCs than control-VSMCs (Figure 2D). Levels of p53 and p21 were higher in MFS-VSMCs than control-VSMCs (Figure 2E). Because a key feature of senescent cells is the SASP, we used ELISAs to measure the levels of SASP factors in medium conditioned by MFS- and control-VSMCs. We found that MFS-VSMCs secreted higher levels of IL-6, IL-8, TNF-α and INF-γ than control-VSMCs (Figure 2F). MFS-VSMCs thus exhibit the characteristic features of cellular senescence.

### TGF-β1 induces cellular senescence of VSMCs in MFS

**Figure 3:**
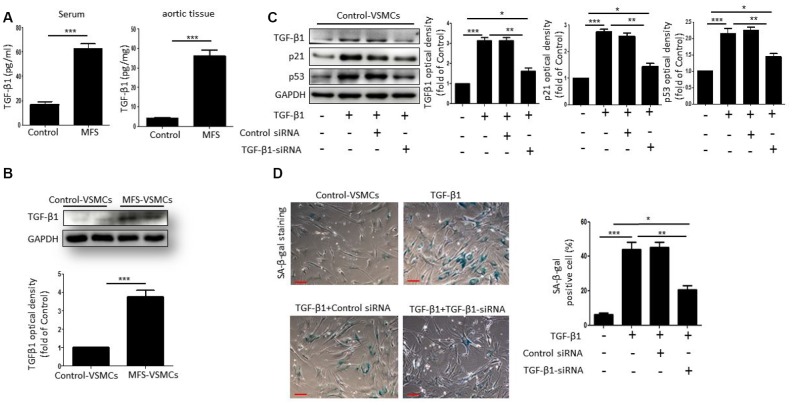
**TGF-β1 induces cellular senescence in VSMCs.** (**A**) TGF-β1 concentrations in serum from control donors and MFS patients was measured using an ELISA. n=6. (**B**) Western blotting and quantitative analysis of TGF-β1 levels in control- and MFS-VSMCs. n=3. (**C**) Western blotting and quantitative analysis of TGF-β1, p53 and p21 levels in control-VSMCs left untreated or treated with TGF-β1 or TGF-β1 combined with control-siRNA or TGF-β1-siRNA. n=3. (**D**) Representative images and quantitative analysis of SA-β-gal staining in control-VSMCs left untreated or treated with TGF-β1 or TGF-β1 combined with control-siRNA or TGF-β1-siRNA. n=3. Numbers of SA-β-gal-positive cells are expressed as percentages of the total cells. Data are expressed as the mean±SEM. *p<0.05, ***p<0.01, ***p<0.001.

To investigate whether TGF-β1 can induce VSMC senescence, we first measured TGF-β1 concentrations in serum and aortic tissue from control donors and MFS patients. Compared with control donors, the level of TGF-β1 was markedly upregulated in both the serum and aortic tissue from MFS patients (Figure 3A). Moreover, western blotting showed that TGF-β1 levels were higher in MFS-VSMCs than control-VSMCs (Figure 3B). To determine whether TGF-β1 induces senescence of VSMCs, we treated control-VSMCs with 50 ng/ml TGF-β1 for 48 h. Subsequent Western blotting showed that TGF-β1-treated control-VSMCs exhibited elevated levels of TGF-β1, p53 and p21, and that this effect was blocked by TGF-β1 knockdown using targeted siRNA (Figure 3C). TGF-β1 treatment also upregulated SA-β-gal activity in control-VSMCs, and that effect, too, was suppressed by TGF-β1 knockdown (Figure 3D). Collectively, these data suggest that VSMC senescence is induced by TGF-β1.

### TGF-β1 induces VSMC senescence through ROS generation

**Figure 4:**
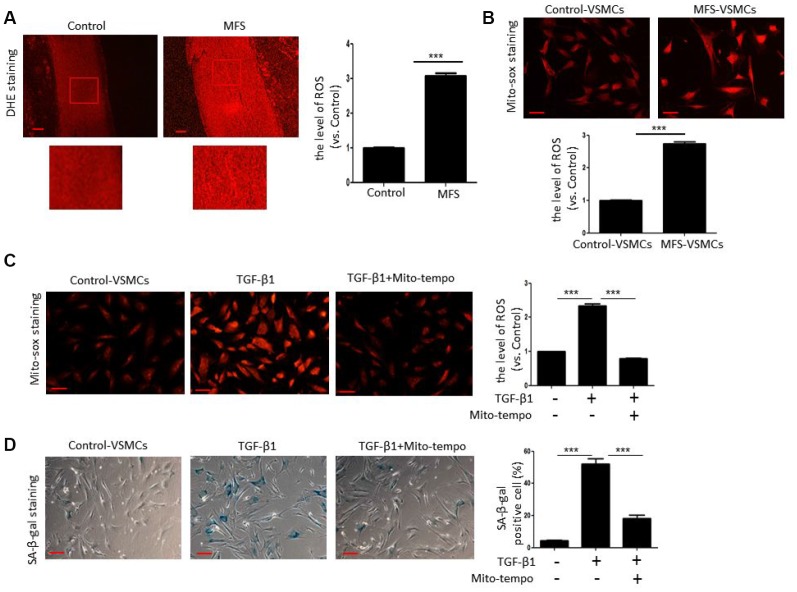
**TGF-β1 induces cellular senescence in VSMCs through elevation of ROS.** (**A**) Representative images and quantitative analysis of DHE staining in the ascending aorta of control donors and MFS patients. ROS levels were analyzed and expressed relative to control. Scale bar=200 μm. n=6. (**B**) Representative images and quantitative analysis of Mito-sox staining of control- and MFS-VSMCs. ROS levels were analyzed and expressed relative to control. n=3. Scale bar=100 μm. (**C**) Representative images and quantitative analysis of Mito-sox staining in control-VSMCs left untreated or treated with TGF-β1 or TGF-β1 combined with Mito-tempo. ROS levels was analyzed and expressed relative to control. n=3. Scale bar=100 μm. (**D**) Representative images and quantitative analysis of SA-β-gal staining in control-VSMCs left untreated or treated with TGF-β1 or TGF-β1 combined with Mito-tempo. n=3. Scale bar=100 μm. Numbers of SA-β-gal-positive cells are expressed as percentages of the total cells. Data are expressed as the mean±SEM. ***p<0.001.

Next, we tested whether TGF-β1 induces cellular senescence of VSMCs through ROS generation. We first examined ROS generation in aortic tissue from control donors and MFS patients using DHE staining. Compared with control donors, ROS levels were significantly elevated in aortic tissue from MFS patients (Figure 4A). In addition, Mito-sox staining revealed that mitochondrial ROS levels were much higher in MFS-VSMCs than control-VSMCs (Figure 4B). We also observed that that both ROS generation (Figure 4C) and SA-β-gal activity (Figure 4D) were greatly enhanced in control-VSMC treated with TGF-β1. This suggests TGF-β1-induced ROS generation likely contributes to the development of VSMC senescence. Consistent with that idea, Mito-tempo, a mitochondria-targeted antioxidant, effectively inhibited ROS production (Figure 4C) and diminished SA-β-gal activity (Figure 4D) in TGF-β1-treated control-VSMCs. These data indicate that TGF-β1 induces VSMC senescence at least in part via ROS generation.

### ROS-activating NF-κB is involved in TGF-β1-induced VSMC senescence

**Figure 5:**
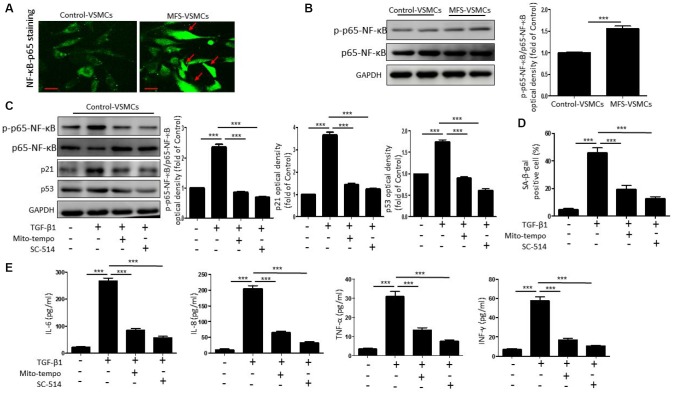
**TGF-β1 induces cellular senescence in VSMCs through activation of ROS/NF-κB signaling.** (**A**) Representative images of p65-NF-κB staining in control- and MFS-VSMCs. Scale bar=50 μm. (**B**) Western blotting and quantitative analysis of p-p65-NF-κB and p65-NF-κB levels in control- and MFS-VSMCs. (**C**) Western blotting and quantitative analysis of p-p65-NF-κB, p65-NF-κB, p53 and p21 levels in control-VSMCs treated with TGF-β1, TGF-β1+Mito-tempo or TGF-β1+SC-154. (**D**) Quantitative analysis of SA-β-gal staining in control-VSMCs treated with TGF-β1, TGF-β1+Mito-tempo or TGF-β1+SC-154. (**E**) Concentrations of IL-6, IL-8, TNF-α and INF-γ in medium conditioned by control-VSMCs treated with TGF-β1, TGF-β1+Mito-tempo or TGF-β1+SC-154. Data are expressed as the mean±SEM. n=3. ***p<0.001.

Because NF-κB is known to promote cellular senescence and SASP secretion [16], we investigated whether TGF-β1 induces VSMC senescence through ROS-mediated activation of the NF-κB signaling pathway. We first used immunofluorescent staining to examine the nuclear translocation of p65-NF-κB, an index of NF-κB activation, in control- and MFS-VSMCs. We observed that translocation of p65-NF-κB from the cytoplasm to the nucleus was greater in MFS- VSMCs than control-VSMCs (Figure 5A), indicating greater NF-kB activation in MFS-VSMCs. Levels of phosphorylated (p)p65-NF-κB were also significantly higher in MFS- than control-VSMCs (Figure 5B). In addition, TGF-β1 treatment upregulated expression of p-p65-NF-κB, p53, and p21 (Figure 5C) and enhanced SA-β-gal activity (Figure 5D) in control-VSMCs. All these effects significantly inhibited by Mito-tempo or the NF-κB inhibitor SC-154 (Figure 5C, 5D). This suggests TGF-β1 mediates NF-κB activation through ROS generation, which in turn promotes VSMC senescence. Mito-Tempo or SC-154 also inhibited the TGF-β1-induced increases in IL-6, IL-8, TNF-α and INF-γ release from control-VSMCs (Figure 5E). These results show that TGF-β1 induced the SASP via a ROS/NF-κB pathway in VSMCs.

## DISCUSSION

**Figure 6:**
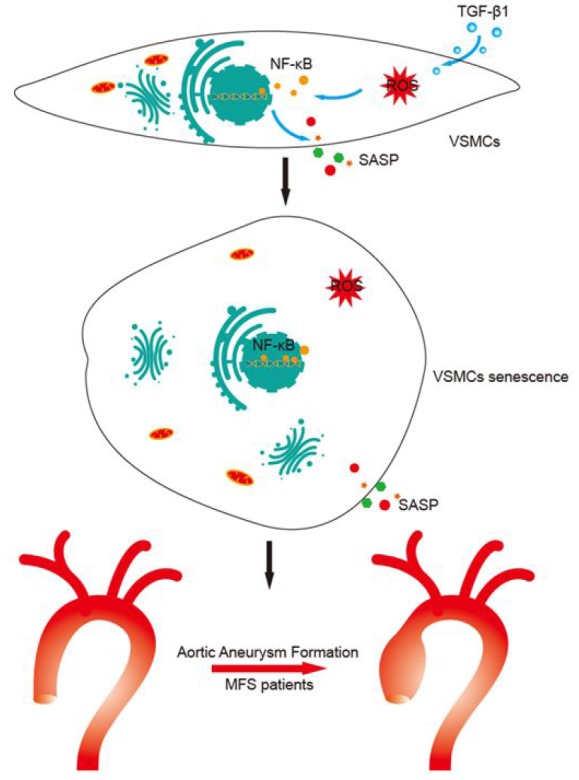
**Proposed mechanisms for TGF-β1-induced VSMC senescence.** This study shows that TGF-β1 induces VSMC senescence through activation of ROS/NF-κB signaling, which leads to aortic aneurysm formation in MFS patients.

There were several major findings of the current study (Figure 6). First, VSMCs within aortic aneurysms in MFS patients exhibited cellular senescence *in vitro* and *in vivo*. Second, upregulated TGF-β1 levels lead to VSMC senescence by promoting mitochondrial ROS generation. Third, induction of VSMC senescence and SASP secretion is mediated via a ROS-activated NF-κB signaling pathway. Based on these results, we conclude that TGF-β1 induces VSMC senescence via the ROS/NF-κB signaling pathway in patients with MFS.

Despite the recent advances in pharmacological therapy and surgery, aortic aneurysm or dissection remains a potentially lethal complication of MFS. This is in large part because their pathogenesis remains unclear. The pathological changes in the tunica media of the aortic wall are closely associated with aortic aneurysm or dissection [17]. This layer is mainly composed of elastic fibers and VSMCs, which suggests VSMC dysfunction likely underlies aortic aneurysm or dissection in MFS patients. Consistent with that idea, alteration of the VSMC phenotype reportedly contributes to aortic aneurysm formation or dissection in MFS [1, 18]. It has been reported that VPO1 promotes VSMC phenotypic switching through activation of the HOCl/ERK1/2 signaling pathway with consequent development of aortic aneurysm [19]. The XBP1u-FoxO4-myocardin axis is essential for maintaining the VSMC phenotype and blocking signaling leading to VSMC phenotypic transition [20]. VSMC senescence is another major cause of VSMC phenotypic changes [21]. Senescent VSMCs release matrix metalloproteinase-9 (MMP9) and secret various SASP factors, including multiple inflammatory cytokines and ECM-degrading proteins, which leads to disruption of tissue structure and reduced function [22, 23]. In the present study, α-SMA^+^p53^+^ double positivity and SA-β-gal activity confirmed the presence of senescent VSMCs within the medial layer of aortic aneurysm tissue from MFS patients. Moreover, VSMCs isolated from MFS patients exhibited increased cell size, reduced proliferative capacity (ki-67 positivity), and enhanced SA-β-gal activity. Notably, MFS-VSMCs secreted high levels of the classic SASP cytokines including IL-6 and IL-8. Nonetheless, the potential mechanisms underlying MFS-VSMC senescence have not yet been clarified.

Although the pathogenesis of MFS is not fully understood, it is known that FBN1 gene mutation, the leading cause of MFS, results in TGF-β activation [24]. Indeed, high circulating levels of TGF-β are detected in MFS patients, suggesting TGF-β plays a crucial role in MFS [25]. We also observed elevated TGF-β1 levels in serum and aneurysmal tissue from MFS patients, and recent studies indicate that excessive TGF-β can lead to senescence in several cell types [26, 27]. For example, elevated TGF-β in corneal endothelial cells induces senescence through upregulation of mitochondrial ROS generation, and a mitochondrial ROS scavenger was able to reverse that effect [28]. In the present study, TGF-β1 levels were much higher in MFS- than control-VSMCs, suggesting TGF-β1 contributes to MFS-VSMC senescence. Consistent with that idea, TGF-β1 greatly increased SA-β-gal activity in control-VSMCs, and TGF-β1 knockdown using siRNA significantly inhibited that response. TGF-β1 also enhanced ROS generation in VSMCs, and that effect was blocked by Mito-tempo, which suggests TGF-β1 induces VSMC senescence by stimulating ROS generation.

Previous studies showed that NF-κB activation triggers cellular senescence [29, 30]. In addition, NF-κB stimulates the pro-inflammatory arm of the SASP, leading to secretion of proinflammatory cytokines [31]. Membrane-bound CD40L promotes lung adenocarcinoma cell senescence and stimulates SASP through activation of NF-κB, while NF-κB knockdown partially those effects [16]. Here, we found that translocation of p65-NF-κB to the nucleus and its phosphorylation were greatly increased in MFS-VSMCs compared to control-VSMCs. This suggests NF-κB activation may be associated with VSMC senescence. ROS can activate NF-κB to induce cellular senescence and the SASP, while NF-κB activation stimulates ROS generation, thereby forming a ROS/NF-κB loop to induce cellular senescence [31]. We also found that inhibiting ROS formation or NF-κB signaling attenuated TGF-β1-induced VSMC senescence and blocked induction of the SASP. This suggests TGF-β1 induces VSMC senescence and triggers the SASP in part via a ROS/NF-κB signaling pathway.

There were several limitations to the present study. First, the dose of TGF-β1 was adapted from our earlier study [32]; whether TGF-β1-induced VSMC senescence is a dose-dependent effect has not been determined. Second, the mechanisms underlying TGF-β1-induced mitochondrial ROS generation were not demonstrated. Previous studies have shown that disrupting the mitochondrial dynamic balance leads to ROS generation [33]. Whether TGF-β1 stimulates ROS generation by modulating mitochondrial dynamics in VSMCs remains to be investigated. Third, whether TGF-β1 also induces telomere shortening or abnormal autophagy to promote VSMC senescence requires further investigation. Fourth, whether current pharmacological strategies, such as atenolol or losartan administration, can mitigate the VSMC senescence in patients of MFS requires further investigation. Finally, NF-κB is not the only transcription factor with the ability to activate the SASP. Whether ROS can activate other transcription factors or signaling pathways to induce the SASP warrants further investigation.

In summary, our study shows that excessive TGF-β1 can induce VSMC senescence and initiate the SASP via the ROS/NF-κB signal pathway, leading to aortic aneurysm formation in patients of MFS. This study thus provides new insight into the potential pathogenesis of aortic aneurysm and provides a novel therapeutic target for MFS treatment.

## MATERIALS AND METHODS

### Isolation, culture and characterization of VSMCs

Ascending aortic aneurysm tissue was harvested from MFS patients who underwent surgery to repair the lesion. Samples of healthy human ascending aortic tissue were collected from donors and served as the control group. All procedures were approved by the research ethics board of Guangdong Provincial People's Hospital. Written informed consent was obtained from each study subject. The demographic characteristics of the study patients were described in our previous study [32]. Human VSMCs were isolated from aortic tissues as previously described [1]. Briefly, after cleaning away adipose tissue and washing with PBS, the medial tissue was dissected from the adventitia and intima. Next, the media was cut into 1-2 mm^3^ pieces and transferred to 10-cm poly-L-lysine coated culture plates and incubated for adhesion at 37°C for 1 h. Once attached to the plate, the medial pieces were gently cultured with Dulbecco’s modified Eagle medium (DMEM; Sigma-Aldrich) supplemented with 20% fetal bovine serum (FBS; Gibco) and 100 μg/mL penicillin and streptomycin. The medium was carefully changed every 3 days. VSMCs migrated out from the pieces within 1-2 weeks. The media pieces were then removed and the cells were collected and passaged. VSMCs were identified based on immunofluorescent staining with antibodies against alpha smooth muscle actin (a-SMA) and calponin. All VSMCs used in this study were at passage 2–3.

### HE staining

Aneurysmal tissue from MFS patients and healthy aortic tissue from control donors were fixed with 10% formalin, embedded in paraffin, cut into 5-μm-thick sections, and mounted on slides. The sections were then stained with hematoxylin and eosin (HE) using the standard protocol.

### DHE staining

ROS levels within aneurysmal tissue were determined through dihydroethidium (DHE) staining (Thermo Fisher Scientific, D1168). Briefly, the sections were first incubated with 10 μM DHE for 30 min at room temperature. They were then washed with PBS and the fluorescent signal was photographed in randomly selected areas using a motorized inverted microscope. The fluorescence intensity was analyzed using Image J software.

### Senescence-associated β-galactosidase (SA-β-gal) staining

VSMC senescence was assessed based on SA-β-gal staining according to the manufacturer’s protocol (Beyotime, C0602). Briefly, control VSMCs and MFS VSMCs were plated in a 6-well culture plate. Some of the control cells were treated for 48 h with 50 ng/ml TGF-β1 (PeproTech, 100-21) combined with 100 μM Mito-tempo (Santa Cruz, SC-221945) or with 1 μM SC-54, a NF-κB inhibitor (Sigma-Aldrich, SML0557). The cells were then washed with PBS, fixed for 30 mins, and stained with SA-β-gal staining solution over night at 37°C (without CO_2_). After washing three times with PBS, the cells were randomly photographed.

### Mito-sox staining

Mitochondrial ROS levels in VSMCs were measured based on Mito-sox staining. Briefly, VSMCs were cultured in 24-well plates before treatment. The VSMCs were next washed with PBS and incubated with 10 μM Mito-sox (Invitrogen, M36008) for 10 min at 37°C in the dark. The cells were then washed again with PBS, and the fluorescence signal was photographed in randomly selected areas using a motorized inverted microscope (Olympus, Hamburg, Germany). Finally, for each group, the fluorescence intensity in five selected microscope fields in three independent experiments were determined using Image J.

### TGF-β1 silencing using small-interfering RNA (siRNA)

VSMCs were transfected with TGF-β1-siRNA (Santa Cruz, SC-44146) or control siRNA (Santa Cruz, SC-37007) using a Lipofectamine RNAiMAX Reagent Kit (Invitrogen, 13778030) according to the manufacturer’s protocol. Western blotting was used to assess transfection efficiency 72 h after transfection.

### Immunofluorescent staining

For immunofluorescent staining, VSMCs were cultured on cover slips in 24-well plates, after which they were fixed in 4% PFA for 30 min and permeabilized in 0.1% Triton X-100 in PBS for 30 mins. The cells were then incubated with the following primary antibodies overnight at 4°C: anti-α-SMA (1:100, Abcam, ab5694), anti-calponin (1:100, Abcam, ab46794) and anti-ki-67 (1:100, Abcam, ab15580). Thereafter, the cells were incubated with fluorescently-labeled secondary antibodies (1:1000) for 1 h at room temperature. The labeled VSMCs were washed with PBS and counterstained with 4′, 6-diamidino-2-phenylindole (DAPI), after which the fluorescent signal from randomly selected areas was photographed using a fluorescence microscope.

### Western blotting

The proteins were extracted using RIPA buffer (CST, 9806), after which their concentration measured using a BCA assay kit (Thermo, 231227). Aliquots of lysate containing 30 μg of protein were then subjected to SDS/PAGE, and the separated proteins were transferred to a PVDF membrane. After blocking with 5% fat-free milk in TBST, the membrane was incubated overnight at 4°C with the primary antibodies: anti-TGF-β1 (Abcam, ab64715), anti-p53 (Abcam, ab26), anti-p21 (Abcam, ab109199) and anti- GAPDH (CST, 2118). The membrane was then washed three times with TBST and incubated with secondary antibodies (1:3000, CST) for at least 1 h at room temperature and exposed in a dark room.

### Analysis of the secretory phenotype

Conditioned medium from VSMCs was prepared as previously described [34]. The concentrations of SASP-related cytokines, including IL-6, IL-8, INF-γ and TNF-α, in the conditioned medium was assessed using ELISAs. Each experiment was repeated three times.

### Statistical analysis

All statistical analyses were performed by Prism 5.04 Software (GraphPad Software for Windows, San Diego, CA, USA). Data are presented as the mean±SEM. Comparisons between two groups was made using unpaired Student’s t-test and between multiple groups using one-way ANOVA followed by Bonferroni test. Values of p <0.05 were considered statistically significant.
